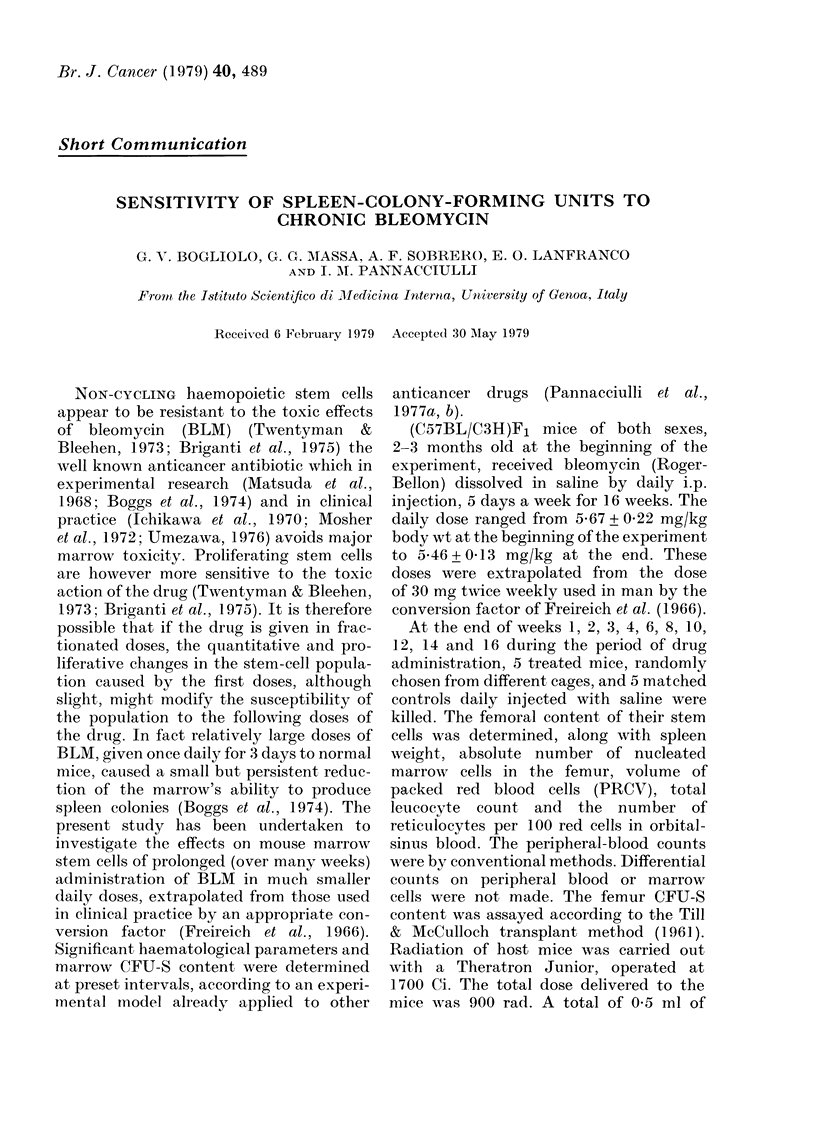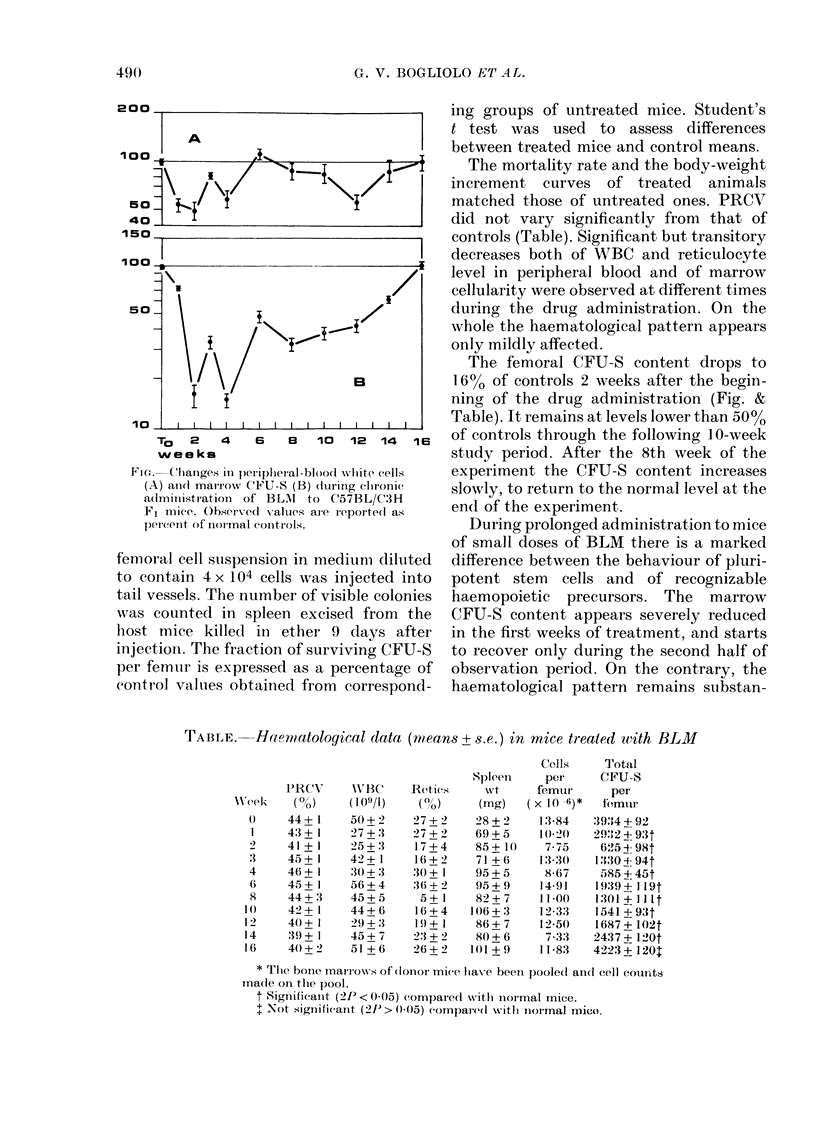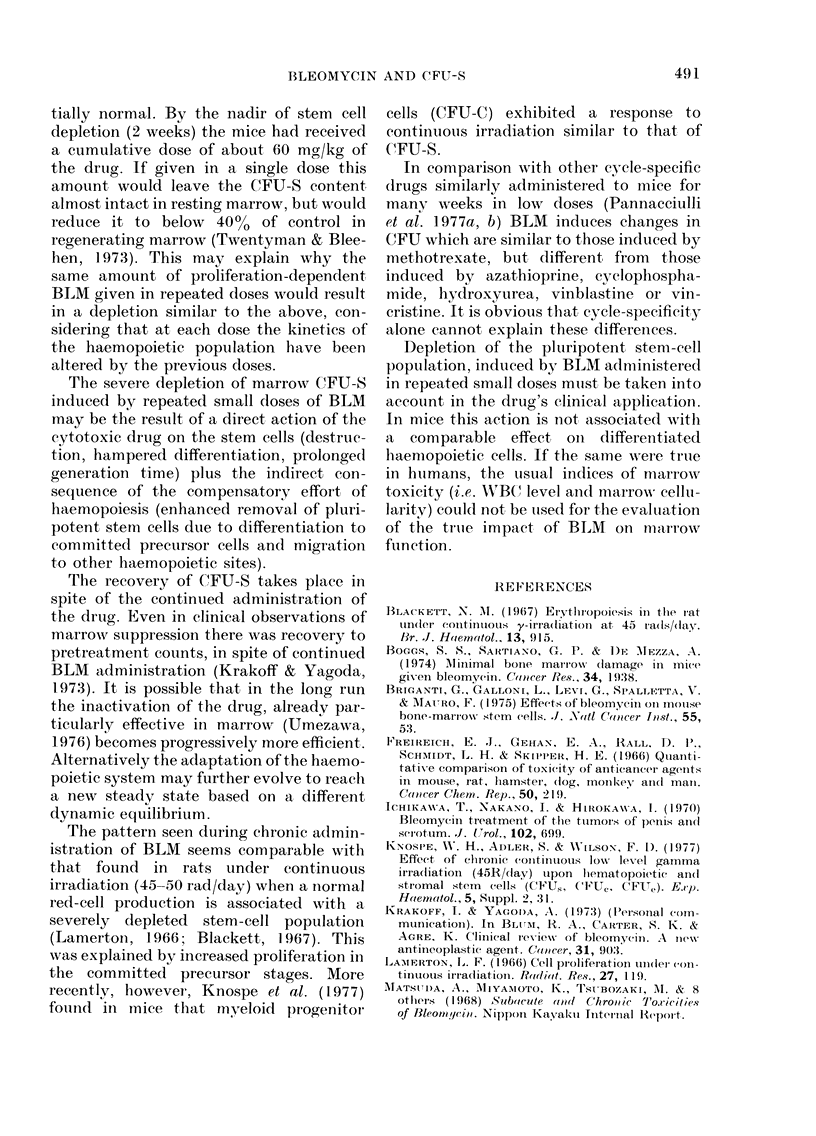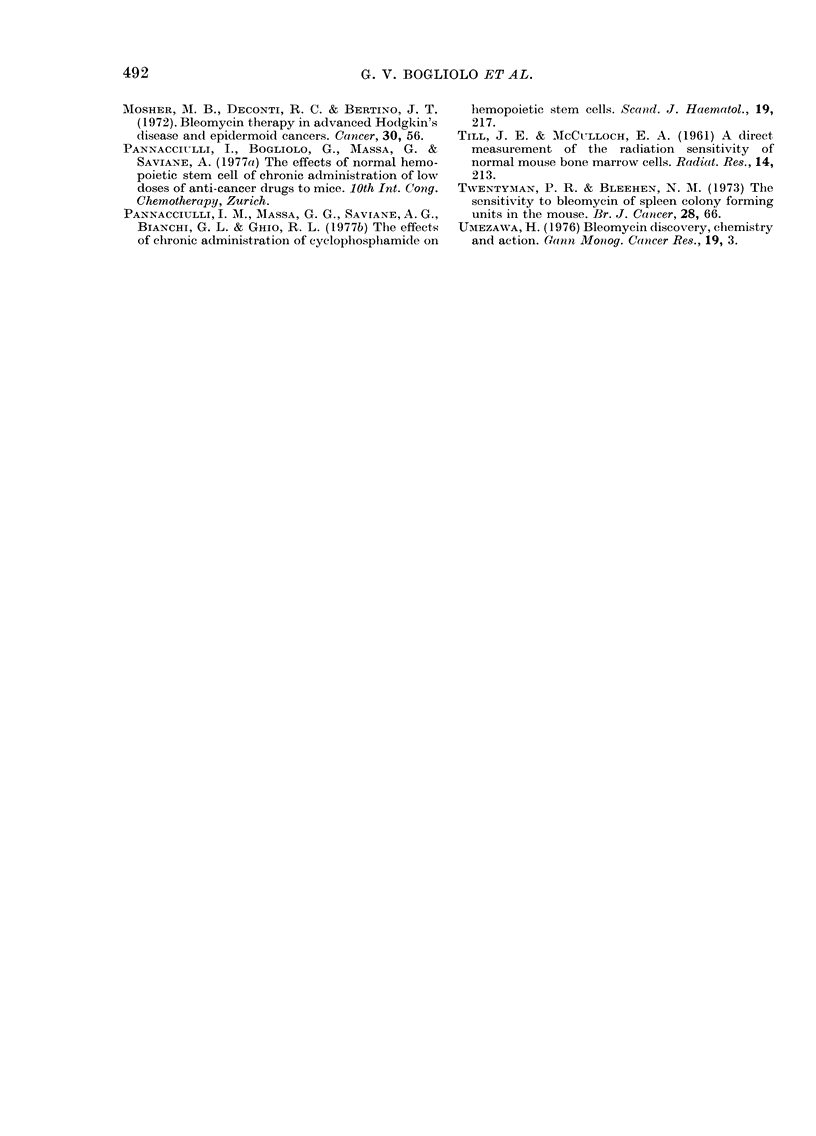# Sensitivity of spleen-colony-forming units to chronic bleomycin.

**DOI:** 10.1038/bjc.1979.205

**Published:** 1979-09

**Authors:** G. V. Bogliolo, G. G. Massa, A. F. Sobrero, E. O. Lanfranco, I. M. Pannacciulli


*Br. J. Cancer* (1979) **40**, 489

***Short Communication***

### SENSITIVITY OF SPLEEN-COLONY-FORMING UNITS TO CHRONIC BLEOMYCIN

G. V. BOGLIOLO, G. G. MASSA, A. F. SOBRERO, E. O. LANFRANCO AND I. M. PANNACCIULLI

*From the Istituto Scientifico di Medicina Interna, University of Genoa, Italy*

Received 6 February 1979 Accepted 30 May 1979

NON-CYCLING haemopoietic stem cells appear to be resistant to the toxic effects of bleomycin (BLM) (Twentyman & Bleehen, 1973; Briganti *et al.*, 1975) the well known anticancer antibiotic which in experimental research (Matsuda *et al.*, 1968; Boggs *et al.*, 1974) and in clinical practice (Ichikawa *et al.*, 1970; Mosher *et al.*, 1972; Umezawa, 1976) avoids major marrow toxicity. Proliferating stem cells are however more sensitive to the toxic action of the drug (Twentyman & Bleehen, 1973; Briganti *et al.*, 1975). It is therefore possible that if the drug is given in fractionated doses, the quantitative and proliferative changes in the stem-cell population caused by the first doses, although slight, might modify the susceptibility of the population to the following doses of the drug. In fact relatively large doses of BLM, given once daily for 3 days to normal mice, caused a small but persistent reduction of the marrow's ability to produce spleen colonies (Boggs *et al.*, 1974). The present study has been undertaken to investigate the effects on mouse marrow stem cells of prolonged (over many weeks) administration of BLM in much smaller daily doses, extrapolated from those used in clinical practice by an appropriate conversion factor (Freireich *et al.*, 1966). Significant haematological parameters and marrow CFU-S content were determined at preset intervals, according to an experimental model already applied to other anticancer drugs (Pannacciulli *et al.*, 1977*a, b*).

(C57BL/C3H)$F_1$ mice of both sexes, 2–3 months old at the beginning of the experiment, received bleomycin (Roger-Bellon) dissolved in saline by daily i.p. injection, 5 days a week for 16 weeks. The daily dose ranged from $5{\cdot}67 \pm 0{\cdot}22$ mg/kg body wt at the beginning of the experiment to $5{\cdot}46 \pm 0{\cdot}13$ mg/kg at the end. These doses were extrapolated from the dose of 30 mg twice weekly used in man by the conversion factor of Freireich *et al.* (1966).

At the end of weeks 1, 2, 3, 4, 6, 8, 10, 12, 14 and 16 during the period of drug administration, 5 treated mice, randomly chosen from different cages, and 5 matched controls daily injected with saline were killed. The femoral content of their stem cells was determined, along with spleen weight, absolute number of nucleated marrow cells in the femur, volume of packed red blood cells (PRCV), total leucocyte count and the number of reticulocytes per 100 red cells in orbital-sinus blood. The peripheral-blood counts were by conventional methods. Differential counts on peripheral blood or marrow cells were not made. The femur CFU-S content was assayed according to the Till & McCulloch transplant method (1961). Radiation of host mice was carried out with a Theratron Junior, operated at 1700 Ci. The total dose delivered to the mice was 900 rad. A total of 0·5 ml of

FIG.—Changes in peripheral-blood white cells (A) and marrow CFU-S (B) during chronic administration of BLM to C57BL/C3H $F_1$ mice. Observed values are reported as percent of normal controls.

femoral cell suspension in medium diluted to contain $4 \times 10^4$ cells was injected into tail vessels. The number of visible colonies was counted in spleen excised from the host mice killed in ether 9 days after injection. The fraction of surviving CFU-S per femur is expressed as a percentage of control values obtained from corresponding groups of untreated mice. Student's $t$ test was used to assess differences between treated mice and control means.

The mortality rate and the body-weight increment curves of treated animals matched those of untreated ones. PRCV did not vary significantly from that of controls (Table). Significant but transitory decreases both of WBC and reticulocyte level in peripheral blood and of marrow cellularity were observed at different times during the drug administration. On the whole the haematological pattern appears only mildly affected.

The femoral CFU-S content drops to 16% of controls 2 weeks after the beginning of the drug administration (Fig. & Table). It remains at levels lower than 50% of controls through the following 10-week study period. After the 8th week of the experiment the CFU-S content increases slowly, to return to the normal level at the end of the experiment.

During prolonged administration to mice of small doses of BLM there is a marked difference between the behaviour of pluripotent stem cells and of recognizable haemopoietic precursors. The marrow CFU-S content appears severely reduced in the first weeks of treatment, and starts to recover only during the second half of observation period. On the contrary, the haematological pattern remains substan-

TABLE.—*Haematological data (means ± s.e.) in mice treated with BLM*

| Week | PRCV (%) | WBC ($10^9$/l) | Retics (%) | Spleen wt (mg) | Cells per femur ($\times 10^{-6}$)* | Total CFU-S per femur |
|---|---|---|---|---|---|---|
| 0 | 44 ± 1 | 50 ± 2 | 27 ± 2 | 28 ± 2 | 13·84 | 3934 ± 92 |
| 1 | 43 ± 1 | 27 ± 3 | 27 ± 2 | 69 ± 5 | 10·20 | 2932 ± 93† |
| 2 | 41 ± 1 | 25 ± 3 | 17 ± 4 | 85 ± 10 | 7·75 | 625 ± 98† |
| 3 | 45 ± 1 | 42 ± 1 | 16 ± 2 | 71 ± 6 | 13·30 | 1330 ± 94† |
| 4 | 46 ± 1 | 30 ± 3 | 30 ± 1 | 95 ± 5 | 8·67 | 585 ± 45† |
| 6 | 45 ± 1 | 56 ± 4 | 36 ± 2 | 95 ± 9 | 14·91 | 1939 ± 119† |
| 8 | 44 ± 3 | 45 ± 5 | 5 ± 1 | 82 ± 7 | 11·00 | 1301 ± 111† |
| 10 | 42 ± 1 | 44 ± 6 | 16 ± 4 | 106 ± 3 | 12·33 | 1541 ± 93† |
| 12 | 40 ± 1 | 29 ± 3 | 19 ± 1 | 86 ± 7 | 12·50 | 1687 ± 102† |
| 14 | 39 ± 1 | 45 ± 7 | 23 ± 2 | 80 ± 6 | 7·33 | 2437 ± 120† |
| 16 | 40 ± 2 | 51 ± 6 | 26 ± 2 | 101 ± 9 | 11·83 | 4223 ± 120‡ |

* The bone marrows of donor mice have been pooled and cell counts made on the pool.

† Significant ($2P < 0·05$) compared with normal mice.

‡ Not significant ($2P > 0·05$) compared with normal mice.

tially normal. By the nadir of stem cell depletion (2 weeks) the mice had received a cumulative dose of about 60 mg/kg of the drug. If given in a single dose this amount would leave the CFU-S content almost intact in resting marrow, but would reduce it to below 40% of control in regenerating marrow (Twentyman & Bleehen, 1973). This may explain why the same amount of proliferation-dependent BLM given in repeated doses would result in a depletion similar to the above, considering that at each dose the kinetics of the haemopoietic population have been altered by the previous doses.

The severe depletion of marrow CFU-S induced by repeated small doses of BLM may be the result of a direct action of the cytotoxic drug on the stem cells (destruction, hampered differentiation, prolonged generation time) plus the indirect consequence of the compensatory effort of haemopoiesis (enhanced removal of pluripotent stem cells due to differentiation to committed precursor cells and migration to other haemopoietic sites).

The recovery of CFU-S takes place in spite of the continued administration of the drug. Even in clinical observations of marrow suppression there was recovery to pretreatment counts, in spite of continued BLM administration (Krakoff & Yagoda, 1973). It is possible that in the long run the inactivation of the drug, already particularly effective in marrow (Umezawa, 1976) becomes progressively more efficient. Alternatively the adaptation of the haemopoietic system may further evolve to reach a new steady state based on a different dynamic equilibrium.

The pattern seen during chronic administration of BLM seems comparable with that found in rats under continuous irradiation (45–50 rad/day) when a normal red-cell production is associated with a severely depleted stem-cell population (Lamerton, 1966; Blackett, 1967). This was explained by increased proliferation in the committed precursor stages. More recently, however, Knospe *et al.* (1977) found in mice that myeloid progenitor cells (CFU-C) exhibited a response to continuous irradiation similar to that of CFU-S.

In comparison with other cycle-specific drugs similarly administered to mice for many weeks in low doses (Pannacciulli *et al.* 1977*a*, *b*) BLM induces changes in CFU which are similar to those induced by methotrexate, but different from those induced by azathioprine, cyclophosphamide, hydroxyurea, vinblastine or vincristine. It is obvious that cycle-specificity alone cannot explain these differences.

Depletion of the pluripotent stem-cell population, induced by BLM administered in repeated small doses must be taken into account in the drug's clinical application. In mice this action is not associated with a comparable effect on differentiated haemopoietic cells. If the same were true in humans, the usual indices of marrow toxicity (*i.e.* WBC level and marrow cellularity) could not be used for the evaluation of the true impact of BLM on marrow function.

### REFERENCES

Blackett, N. M. (1967) Erythropoiesis in the rat under continuous $\gamma$-irradiation at 45 rads/day. *Br. J. Haematol.*, **13**, 915.

Boggs, S. S., Sartiano, G. P. & De Mezza, A. (1974) Minimal bone marrow damage in mice given bleomycin. *Cancer Res.*, **34**, 1938.

Briganti, G., Galloni, L., Levi, G., Spalletta, V. & Mauro, F. (1975) Effects of bleomycin on mouse bone-marrow stem cells. *J. Natl Cancer Inst.*, **55**, 53.

Freireich, E. J., Gehan, E. A., Rall, D. P., Schmidt, L. H. & Skipper, H. E. (1966) Quantitative comparison of toxicity of anticancer agents in mouse, rat, hamster, dog, monkey and man. *Cancer Chem. Rep.*, **50**, 219.

Ichikawa, T., Nakano, I. & Hirokawa, I. (1970) Bleomycin treatment of the tumors of penis and scrotum. *J. Urol.*, **102**, 699.

Knospe, W. H., Adler, S. & Wilson, F. D. (1977) Effect of chronic continuous low level gamma irradiation (45R/day) upon hematopoietic and stromal stem cells ($CFU_S$, $CFU_c$, $CFU_e$). *Exp. Haematol.*, **5**, Suppl. 2, 31.

Krakoff, I. & Yagoda, A. (1973) (Personal communication). In Blum, R. A., Carter, S. K. & Agre, K. Clinical review of bleomycin. A new antineoplastic agent. *Cancer*, **31**, 903.

Lamerton, L. F. (1966) Cell proliferation under continuous irradiation. *Radiat. Res.*, **27**, 119.

Matsuda, A., Miyamoto, K., Tsubozaki, M. & 8 others (1968) *Subacute and Chronic Toxicities of Bleomycin.* Nippon Kayaku Internal Report.

MOSHER, M. B., DECONTI, R. C. & BERTINO, J. T. (1972). Bleomycin therapy in advanced Hodgkin's disease and epidermoid cancers. *Cancer*, **30**, 56.

PANNACCIULLI, I., BOGLIOLO, G., MASSA, G. & SAVIANE, A. (1977*a*) The effects of normal hemopoietic stem cell of chronic administration of low doses of anti-cancer drugs to mice. *10th Int. Cong. Chemotherapy, Zurich.*

PANNACCIULLI, I. M., MASSA, G. G., SAVIANE, A. G., BIANCHI, G. L. & GHIO, R. L. (1977*b*) The effects of chronic administration of cyclophosphamide on hemopoietic stem cells. *Scand. J. Haematol.*, **19**, 217.

TILL, J. E. & MCCULLOCH, E. A. (1961) A direct measurement of the radiation sensitivity of normal mouse bone marrow cells. *Radiat. Res.*, **14**, 213.

TWENTYMAN, P. R. & BLEEHEN, N. M. (1973) The sensitivity to bleomycin of spleen colony forming units in the mouse. *Br. J. Cancer*, **28**, 66.

UMEZAWA, H. (1976) Bleomycin discovery, chemistry and action. *Gann Monog. Cancer Res.*, **19**, 3.