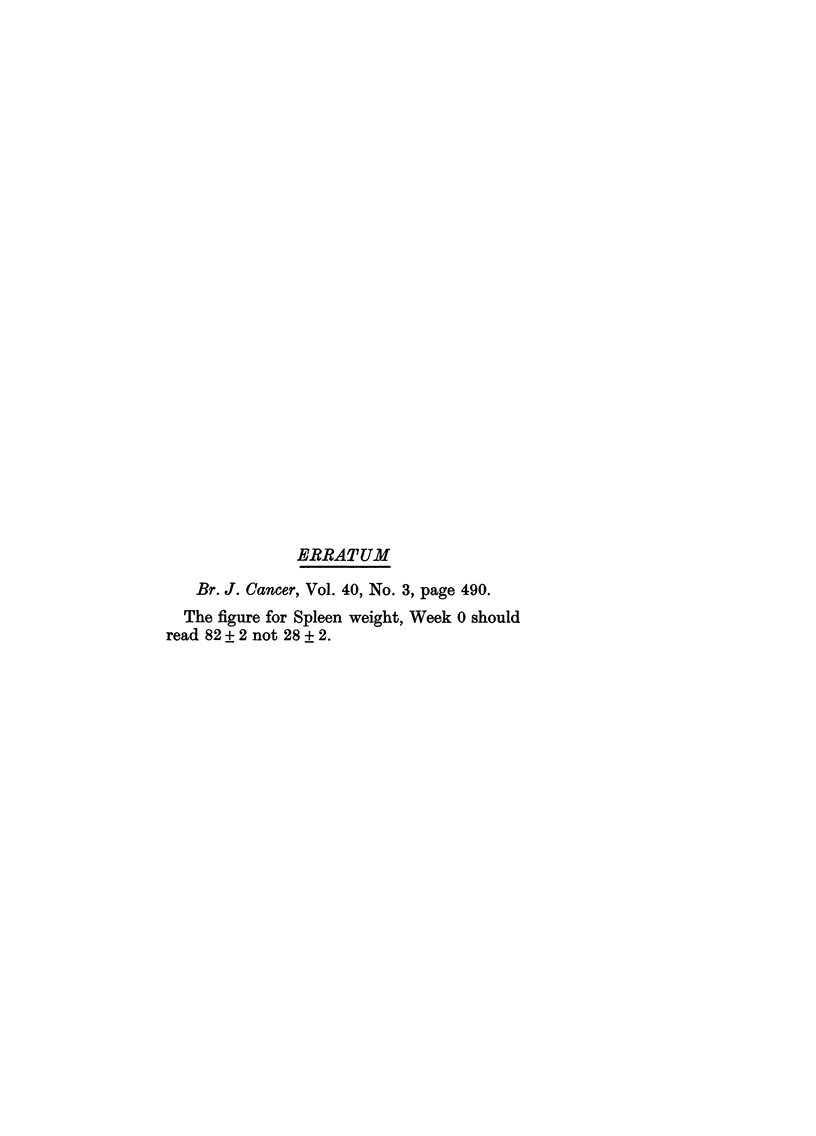# Erratum

**Published:** 1979-09

**Authors:** 


### *ERRATUM*

*Br. J. Cancer*, Vol. 40, No. 3, page 490.

The figure for Spleen weight, Week 0 should read $82 \pm 2$ not $28 \pm 2$.